# Intravascular lesions of the hand

**DOI:** 10.1186/1746-1596-3-24

**Published:** 2008-05-30

**Authors:** Liron Pantanowitz, Wayne H Duke

**Affiliations:** 1 Department of Pathology, Baystate Medical Center, Tufts University School of Medicine, Springfield, MA, USA

## Abstract

**Introduction:**

Intravascular lesions of the hand comprise reactive and neoplastic entities. The clinical diagnosis of such lesions is often difficult, and usually requires pathologic examination. We present the largest series to date of intravascular lesions affecting the hand.

**Methods:**

A retrospective review of intravascular (arterial and venous) lesions involving the hand was conducted. Data regarding clinicopathologic findings were analyzed.

**Results:**

We identified 10 patients with intravascular lesions of their hands including thromboemboli (n = 3), reactive intravascular conditions such as papillary endothelial hyperplasia or Masson's tumor (n = 2) and fasciitis (n = 1), as well as vascular neoplasms including pyogenic granuloma (n = 2) and angioleiomyoma (n = 2).

**Conclusion:**

Blood vessel injury and/or venous thrombosis may predispose to several intravascular lesions of the hand. Recognition of reactive entities from neoplastic conditions is important.

## Background

The hand is a vascular region of the human body containing an extensive network of arteries and veins. These vessels are subject to potential trauma, including iatrogenic injury such as intravenous line placement. Also the hand is a common site for the impaction of emboli, as well as involvement by vascular tumors [1]. The distinction between reactive and neoplastic intravascular lesions of the hand is important. Occasionally, benign intravascular lesions of the hand can be mistaken for malignant neoplasms [2,3].

To date, there have been only a few case reports dealing with intravascular lesions of the hand. Anand et al report a case of intravascular fasciitis in a pregnant woman that affected the hypothenar eminence of her hand associated with the ulnar artery [3]. Rare cases of intravenous pyogenic granuloma (PG) arising within a vein in the palm of a 58-year-old woman [4], and another inside an acquired arterio-venous malformation of the palm of a 44-year-old woman [5], have been reported. Previously, a case of intravascular papillary endothelial hyperplasia arising in the hand of a 23-year-old female fencer was reported [6], raising the possibility of a post-traumatic proliferative histogenesis. To the best of our knowledge, we were unable to identify any large published series dealing with intravascular lesions of the hand. Therefore, the aim of this study was to determine the clinicopathologic findings of intravascular lesions involving the hand in a case series of patients.

## Methods

A 20 year (1987–2007) retrospective review of our pathology archives for reported cases of intravascular (arterial and/or venous) lesions involving the hand and fingers was conducted. Intravascular lesions were defined as pathological lesions located within a blood vessel that partly or completely occluded the lumen. Proliferative (benign and neoplastic) and non-proliferative (thrombi and emboli) entities were included. Cases involving only the microvasculature (capillaries) were excluded. Also, vascular entities (e.g. aneurysms, malformations) without an accompanying intravascular lesion were not included. Data regarding patient demographics (age, gender), comorbid disease, clinical presentation, location (wrist, palm, dorsal hand, finger), size (greatest measurement in cm) and pathologic diagnosis were documented. All available clinical records and histopathologic material were reviewed.

## Results

We identified 10 patients with a confirmed intravascular lesion of their hands (Table 1).

**Table 1:** Patient clinicopathologic characteristics.

Case	Diagnosis	Age (years)	Gender	Presentation	Location	Size (cm)
1	Venous thrombosis	39	Female	Mass	Dorsal hand	2.0
2	Masson's tumor	51	Male	Mass	Palm	0.2
3	Masson's tumor	52	Male	Mass	Finger	0.9
4	Intravascular fasciitis	17	Male	Mass	Wrist	1.2
5	Intravascular pyogenic granuloma	49	Female	Mass	Not specified	0.5
6	Intravascular pyogenic granuloma	36	Male	Mass	Finger	0.7
7	Angioleiomyoma	51	Female	Mass	Finger	2.5
8	Angioleiomyoma	69	Male	Mass	Finger	0.8
9	Arterial thromboemboli	31	Female	Gangrene	Entire hand	0.2
10	Arterial thrombosis	66	Female	Gangrene	Entire hand	0.3

### Clinical findings

There was a M:F ratio of 1:1, and patients were of mean age 46 years old (range, 17 – 69 years). A mass lesion was the presenting symptom in most (80%) cases, occurring on the fingers in at least half of these cases. All mass lesions were surgically excised. One of these patients (case 1) was a 39-year-old female who presented with a mildly tender, palpable, 2.0 cm cord-like mass on the dorsum of her right hand at the site of a recent intravenous line she had in place when she underwent spinal surgery. There was no inflammation on examination and imaging (ultrasound and x-rays) identified no foreign body. We were unable to determine if the remaining cases had a history of hand trauma.

**Figure 1:**
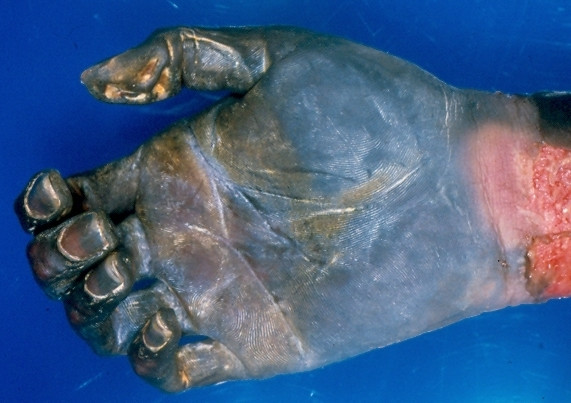
Dry gangrene of an amputated hand due to infarction from septic emboli (case 9).

Two patients presented with significant infarction of their hands. The first patient (case 9) was a 31-year-old intravenous drug addict with AIDS (CD4 count 132 cells/mm^3^) and acute infective endocarditis who developed dry gangrene of her right hand and both feet. She underwent amputations of her right forearm (Figure 1) and both feet. The other patient (case 10) was a 66-year-old female with treated colon cancer undergoing plasmapheresis for suspected thrombocytopenic purpura (TTP) who developed ischemic necrosis of her right hand and bilateral toes, all requiring amputation. Her work-up for vasculitis and hypercoagulability was negative.

### Pathologic findings

**Figure 2:**
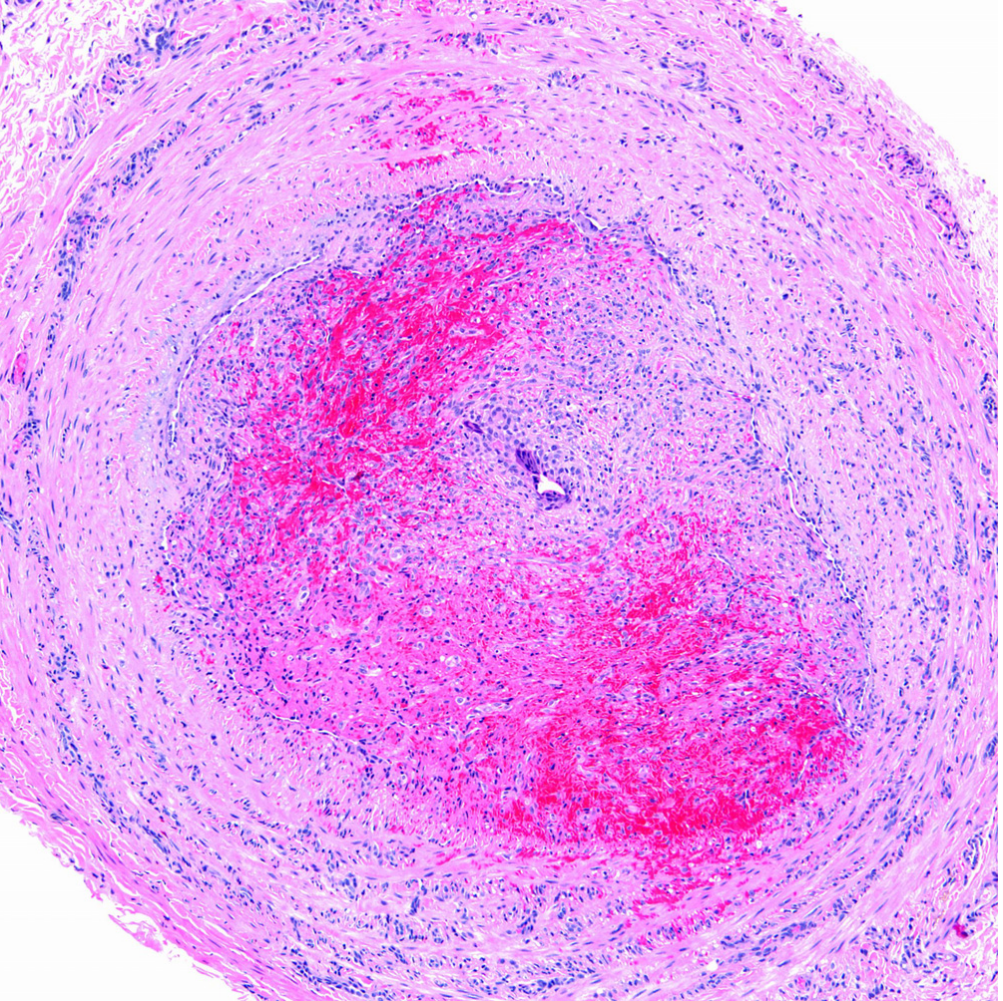
Organizing blood vessel thrombus from case 1 (H&E stain).

**Figure 3:**
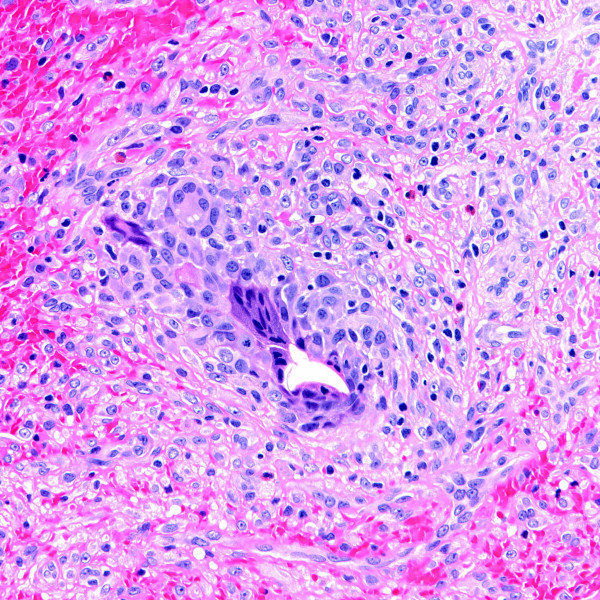
Organizing thrombus with central foreign material and an associated foreign body giant cell reaction seen at higher magnification (H&E stain).

Most (60%) of the intravascular conditions in this series were reactive (i.e. non-neoplastic) in nature, including thrombi (n = 3), Masson's tumor or intravascular papillary endothelial hyperplasia (n = 2), and intravascular fasciitis (n = 1). Mass lesions were of mean size 1.0 cm (range, 0.2 to 2.5 cm). Thrombi (including the impacted emboli in case 9) ranged in size from 0.2 cm to a 2.0 cm long cord-like mass (case 1). In case 1, segmental resection of the patient's dorsal hand vein showed an organizing thrombus containing central, intraluminal refractile foreign material with a surrounding foreign body giant cell reaction (Figures 2 &3). Pathologic examination of the gangrenous hand in case 9 revealed several thromboemboli, including a septic embolus in one of her arteries. In case 10, organizing thrombi were identified occluding atheromatous arteries, accompanied by marked medial calcification. The organizing thrombi showed vessel occlusion with recanalization.

**Figure 4:**
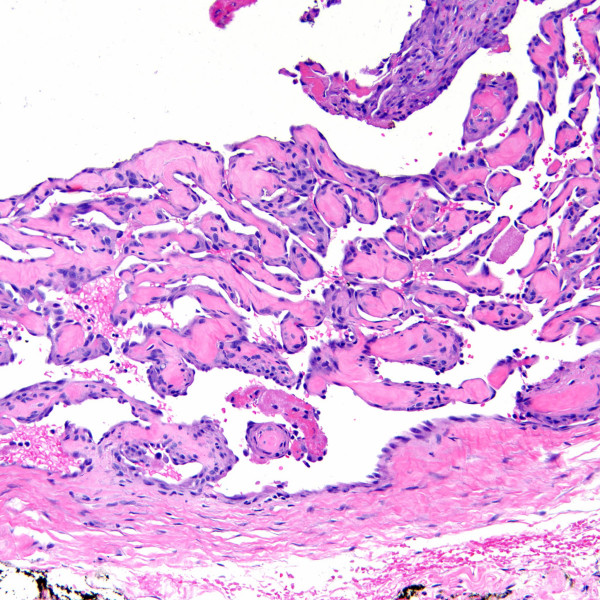
Intravascular papillary endothelial hyperplasia (H&E stain).

**Figure 5:**
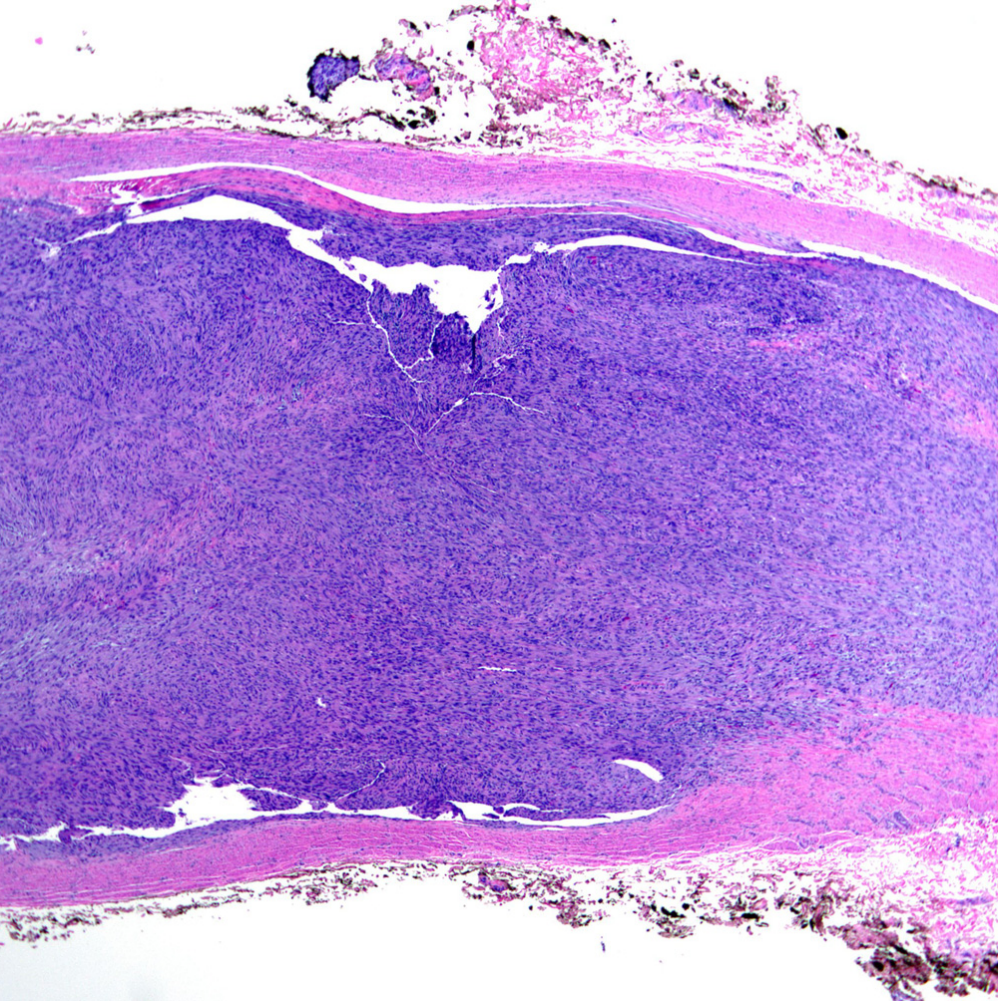
Intravascular fasciitis shown at low power magnification (H&E stain).

**Figure 6:**
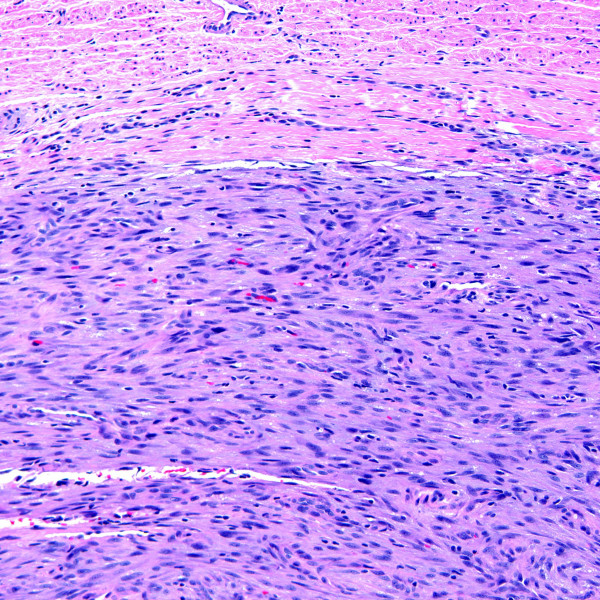
Intravascular fasciitis shown at high power magnification (H&E stain).

**Figure 7:**
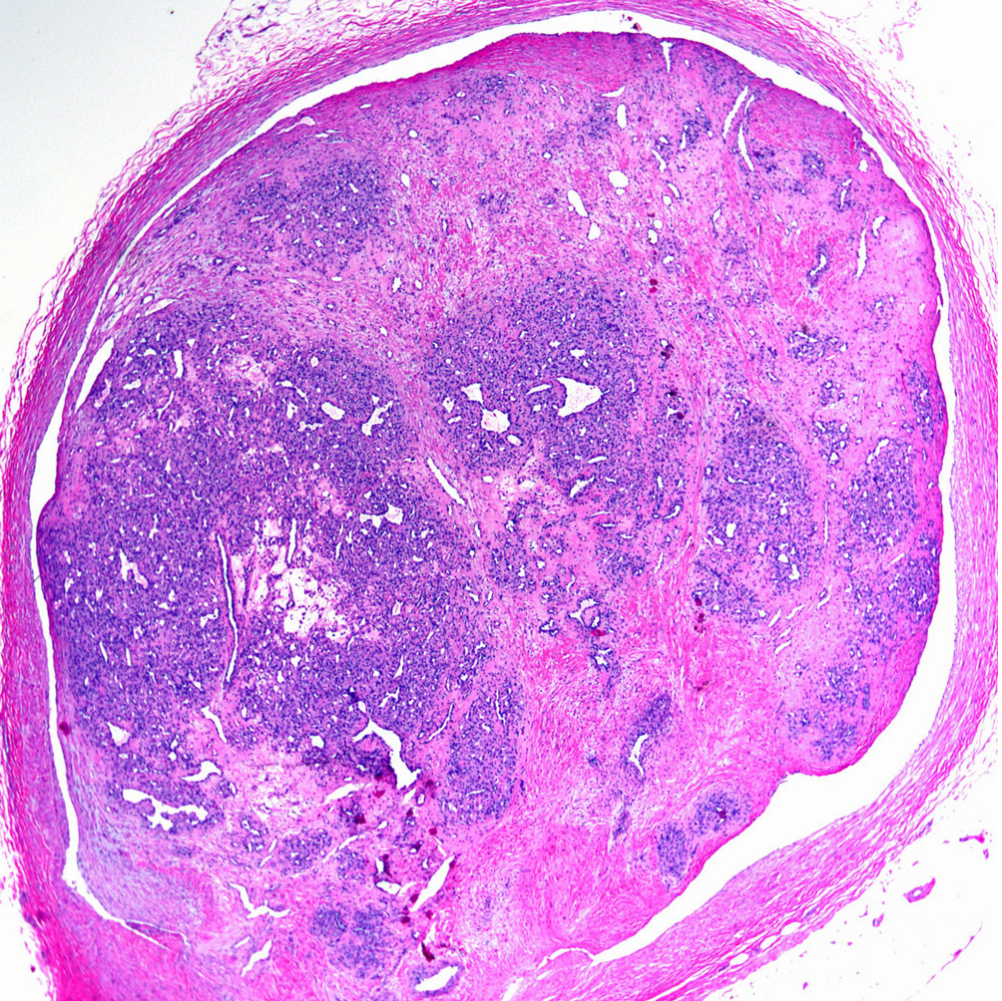
Intravenous pyogenic granuloma (H&E stain).

**Figure 8:**
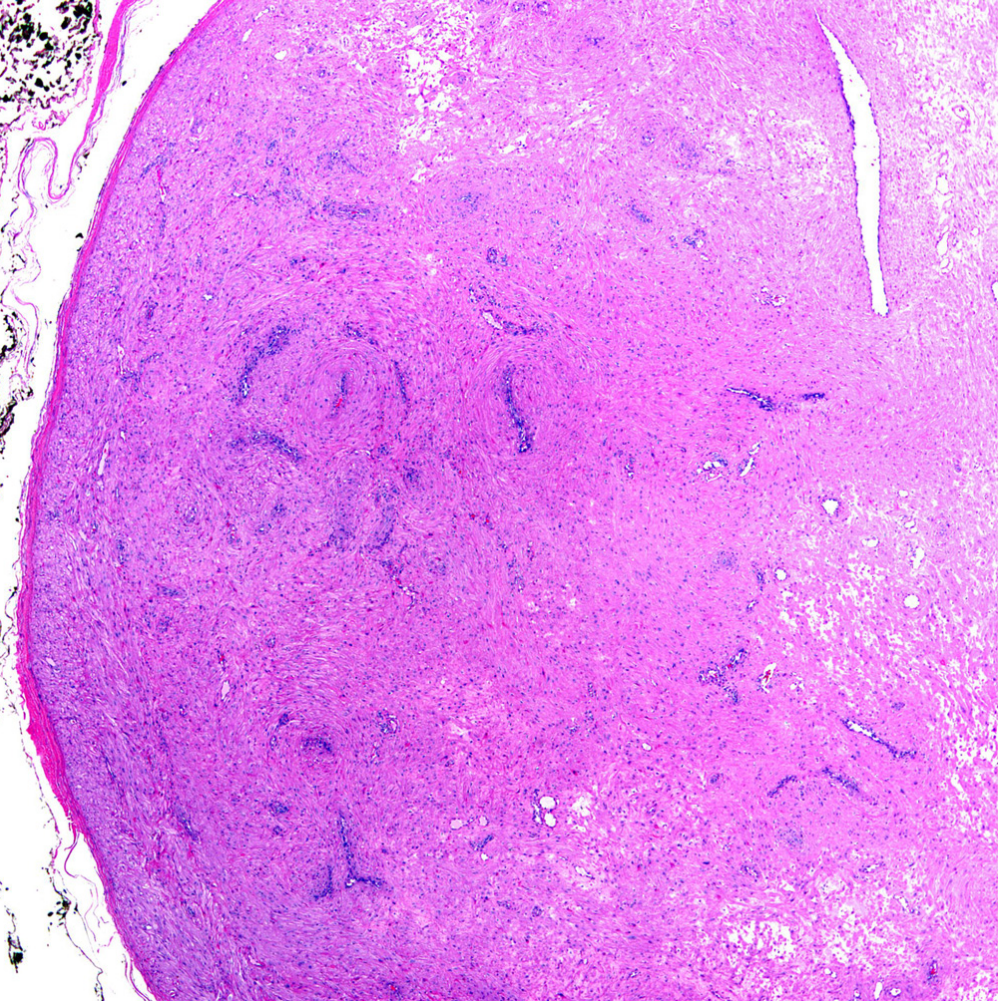
Angioleiomyoma shown at low power magnification (H&E stain).

**Figure 9:**
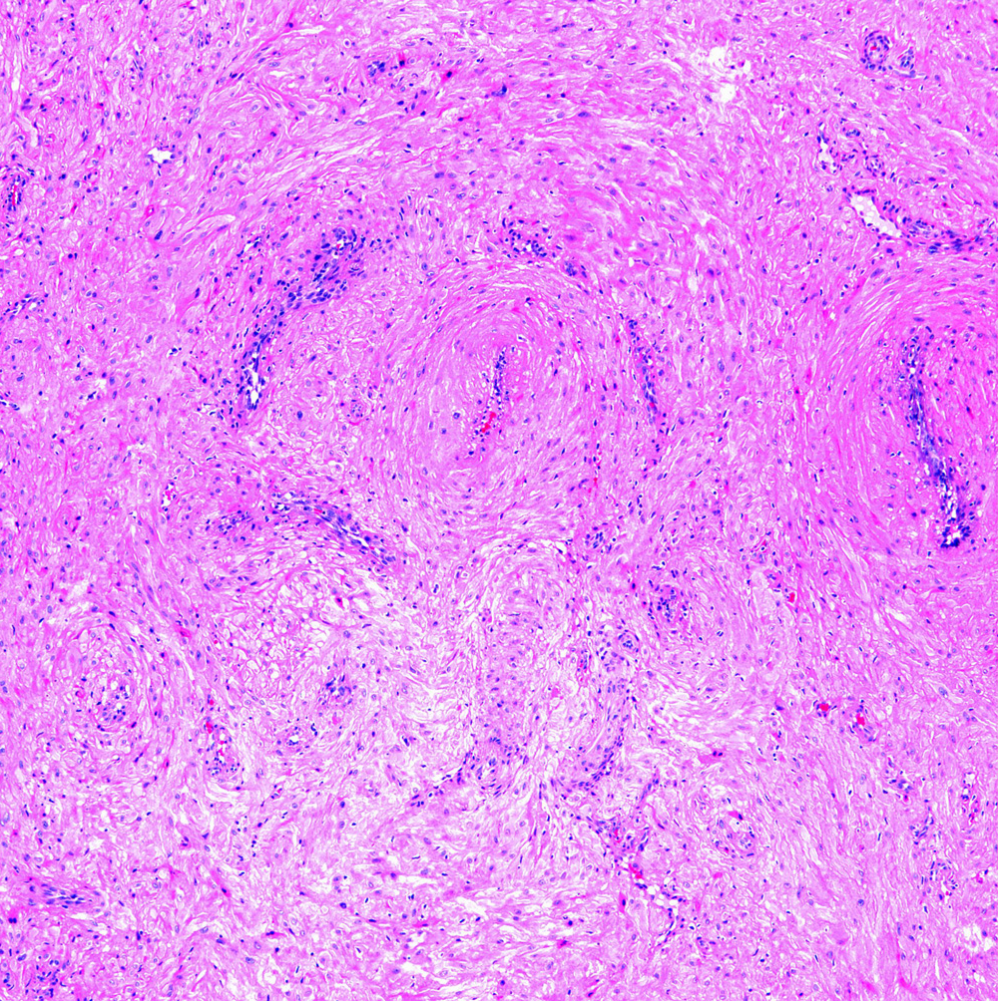
Angioleiomyoma shown at high power magnification (H&E stain).

Intravascular papillary endothelial hyperplasia (Masson's tumors) were characterized by multiple endothelial-lined small papillary structures with hyaline stalks (Figure 4). In one case (case 2) intravascular papillary endothelial hyperplasia was identified partially involving an organizing thrombus within a vein. Intravascular fasciitis (case 4) was identified within a medium-sized muscular vessel, characterized by an intraluminal spindle cell proliferation adherent to the intima (Figures 5 &6). Extravascular involvement was absent. Both cases of intravenous PG developed within the lumen of a vein, and were composed of lobular growths of small, endothelial-lined capillaries (Figure 7). The angioleiomyomas in our series were both of the solid type. They were comprised of closely compacted eosinophilic smooth muscle cells that blended in with admixed muscular blood vessels (Figures 8 &9). Vasculitis and fibromuscular dysplasia of the arteries was not observed. No atypia or features of malignancy were identified in any of the cases.

## Discussion

We present, to the best of our knowledge, the largest series of intravascular lesions involving the hand. As our cases were restricted only to those intravascular lesions submitted to our pathology department for examination, this descriptive study does not provide prevalence data of such entities involving the hand. Nevertheless, despite the fact that our health system includes a pediatric hospital, only adults of both gender were identified in our series. This is surprising, given that vascular lesions like hemangiomas and vascular malformations of the hand are not uncommon in the pediatric population [1]. In our series, most intravascular lesions presented as a palpable mass. Those with thromboembolic disease also displayed features related to blood vessel occlusion (i.e. ischemia and subsequent gangrene). Based upon our data, the differential diagnosis for intravascular lesions of the hand includes organizing thrombus, reactive intravascular conditions (papillary endothelial hyperplasia, and fasciitis), and less likely vascular tumors (PG and angioleiomyoma) that present exclusively or partially as an intravascular process.

There is a paucity of literature pertaining to occlusive thromboembolic disease involving the hands. The etiology of digital vessel occlusion in a prior series was reported to be equally due to emboli (usually from proximal atherosclerotic disease) and thrombi (usually due to collagen vascular disorders) [7]. Pruitt et al, report one case in which a 74-year-old woman, who underwent subclavian and brachiocephalic artery angioplasty with stent placement, developed fatal distal septic emboli involving her one forearm and hand [8]. Emboli that impact in the small arteries of the hand are likely to give rise to serious ischemic effects like infarction. Indeed, one patient in our study with infective endocarditis developed septic emboli that impacted in her hand, with dramatic consequences. In another study involving nine patients with digital amputations secondary to emboli (septic and non-septic) the primary focus of the emboli was usually unknown [9].

In our study, we identified two patients with complete intravascular occlusion due to organizing thrombi, involving a vein in one case and atheromatous artery in another. Factors contributing to thrombus formation may include damage to the endothelium of the vessel wall (as occurred in case 1), alteration in blood flow (e.g. turbulence, stasis or occlusion), and/or the composition of the blood (e.g., a hypercoagulable state such as TTP in our case 10). While thrombi may certainly resolve, some can undergo organization with recanalization due to the ingrowth of fibrous tissue and new capillaries from the wall of the surrounding blood vessel. If recanalization fails to take place, the thrombus can develop into a fibrous scar that permanently obstructs blood flow. They may even calcify to form phleboliths [10]. Obstructive lesions due to fibromuscular intimal proliferation with associated thrombosis and/or distal thromboembolization affecting arteries of the hands and digits is an important lesion not only in the elderly, but also young patients [11].

Intravascular papillary endothelial hyperplasia (Masson's tumor or vegetant intravascular hemangioendothelioma) is a reactive condition representing an exuberant organization and recanalization of a thrombus [12]. The hand and fingers are one of its common locations [13-16]. The diagnosis is based largely upon microscopic examination. As illustrated in our study, this entity is characterized by multiple small papillary structures covered by an attenuated layer of bland appearing endothelium. The papillary cores are made up of fibrin or hyalinized connective tissue. Masson's tumor may occur as a pure (intravascular) form involving a blood vessel, or as a focal change (mixed form) arising in a pre-existing vascular lesion or extravascular organizing hematoma. Both tumors in our study were of the pure form. When they involve a pre-existing vascular neoplasm, the clinical findings are usually those of the latter. They usually occur on the extremities, with a predilection for the fingers [17]. In some cases there can even be numerous lesions, mimicking Kaposi's sarcoma [18]. Simple excision is usually curative, although recurrence has been described [17].

Intravascular fasciitis is a benign, reactive myofibroblastic proliferation arising from the blood vessel wall that can have intraluminal, intramural, and/or extramural involvement of small to medium-sized veins and arteries [19]. Prior trauma, viral infection, venous thrombosis, and possibly pregnancy have all been implicated in the etiology of intravascular fasciitis [3]. As was noted in our one case of intravascular fasciitis (case 4), there appears to be a slight predilection for young males [19,20]. This entity is a distinct variant of nodular fasciitis, which has similar histologic features. They can result in multinodular or serpentine growth along the course of affected blood vessels, presenting as a painless, slowly growing round to oval mass (1.5 to 5 cm in size) [19]. In a prior series reporting on 17 cases of intravascular fasciitis, three (18%) occurred on the hand and one (6%) on the wrist [19]. Rare cases of local recurrence following surgical excision have been reported [19].

Intravenous PG is a rare variant of lobular capillary hemangioma developing within the lumen of a vein. Few case reports of this lesion involving the hand have been published [4,5,21,22]. Some authors regard PG as a hyperplastic process [17]. This is because PG often grows in response to trauma, hormonal factors and/or retinoid therapy, morphologically resembles granulation tissue, and may resolve spontaneously. When PG develops within a vein their attachment to the wall by a stalk can often be identified on pathologic examination. Histologically, they also exhibit a lobular growth pattern of capillaries and venules. However, in the intravascular variant this lobular pattern may not be as pronounced as in their extravascular counterparts [23]. In rare cases, PG lesions may contain a component of a Masson's tumor [24].

Angioleiomyoma (also called angiomyoma or vascular leiomyoma) is a benign tumor that originates in the tunica media of a vein wall. They often present as painful, slow growing subcutaneous nodules on the extremities, including the hand [25]. Imaging studies are often not helpful in distinguishing them from other mass lesions [26]. They are well circumscribed, usually under 2 cm in size, and composed of mature smooth muscle cells which surround and intersect between vascular channels. We included the two cases of solid angioleiomyomas in our series because of their intimate relationship with large vascular channels. A pure intravascular angioleiomyoma may rarely occur, as has been reported by others to occur on the elbow of a 59-year-old man [27]. Based upon their dominant histological pattern, three subtypes are recognized [28]: (i) a solid type with compact muscle bundles and many slit-like vessels, (ii) a venous type with thicker muscular vessels and less compact intervening muscle bundles, and (iii) a cavernous type with more dilated channels and a minor smooth muscle component. Histologically, the smooth muscle bundles appear to extend tangentially ("spin off") from the periphery of the vessels. The spindle cells are immunoreactive for smooth muscle markers (actin and desmin). Simple local excision is adequate treatment, and recurrence is exceptional [28].

Although not identified in our series, the differential diagnosis for intravascular lesions of the hand can be expanded to include intravascular Kaposi's sarcoma, intravascular myopericytoma, intravascular lymphomatosis, and less likely angiolymphoid hyperplasia with eosinophilia (intravenous atypical vascular proliferation) which is considered to represent a late stage of Kimura's disease. Intravascular Kaposi's sarcoma is a newly recognized morphologic variant characterized by an exclusive intravascular growth of interlacing fascicles of human herpesvirus-8, CD31- and CD34-positive spindle cells [29]. It can arise in both Classic (sporadic) and AIDS-related Kaposi's sarcoma, and may present on the hand [29]. Intravascular myopericytoma is a benign tumor, related to angioleiomyoma, comprised of myoid-like spindled cells arranged in a concentric pattern around blood vessels [30]. Intravascular lymphomatosis (or so-called "malignant" angioendotheliomatosis) is an aggressive and rapidly fatal variant of extranodal large B-cell lymphoma seen in adults. It is characterized by the presence of lymphoma cells that occur only in the lumina of small vessels. Cutaneous lesions of intravascular lymphomatosis consist of tender, erythematous or purple nodules and plaques. Frequently afflicted individuals have secondary involvement of their central nervous system and viscera.

## Conclusion

In conclusion, we present the largest series to date of intravascular lesions affecting the hand. The clinical diagnosis of such lesions is difficult, and usually requires pathologic examination. Although the exact pathogenesis for many of these intravascular lesions remains speculative, it is plausible that blood vessel injury and/or venous thrombosis may predispose to their development. Recognition of these various intravascular lesions as benign/reactive entities is important in order to avoid misdiagnosis and potential over-treatment.

## Authors' contributions

LP and WHD contributed equally to all aspects of this study and manuscript. Both authors read and approved the final manuscript.
